# Invasions, DNA barcodes, and rapid biodiversity assessment using ants of Mauritius

**DOI:** 10.1186/1742-9994-6-31

**Published:** 2009-12-10

**Authors:** M Alex Smith, Brian L Fisher

**Affiliations:** 1 Biodiversity Institute of Ontario, Department of Integrative Biology, University of Guelph, Guelph Ontario, N1G 2W1, Canada; 2 Department of Entomology, California Academy of Sciences, San Francisco, USA

## Abstract

**Background:**

Using an understudied taxon (Hymenoptera, Formicidae) found on a tropical island (Mauritius) where native flora and fauna have been threatened by 400 years of habitat modification and introduced species, we tested whether estimated incidences of diversity and complementarity were similar when measured by standard morphological alpha-taxonomy or phylogenetic diversity (PD) based on a standardized mitochondrial barcode and corroborating nuclear marker.

**Results:**

We found that costs related to site loss (considered loss of evolutionary history measured as loss of barcode PD) were not significantly different from predictions made either a) using standard morphology-based taxonomy, or b) measured using a nuclear marker. Integrating morphology and barcode results permitted us to identify a case of initially morphologically-cryptic variation as a new and endemic candidate species. However, barcode estimates of the relative importance of each site or network of sites were dramatically affected when the species in question was known to be indigenous or introduced.

**Conclusion:**

This study goes beyond a mere demonstration of the rapid gains possible for diversity assessment using a standardized DNA barcode. Contextualization of these gains with ecological and natural history information is necessary to calibrate this wealth of standardized information. Without such an integrative approach, critical opportunities to advance knowledge will be missed.

## Background

Life on our planet is disappearing at the highest recorded rate outside of accepted mass extinction events [1,2]. This crisis is exacerbated in insular habitats, where endemic taxa are exposed not only to the competing effects of habitat destruction, fragmentation and degradation, but also to biological invasions that replace native species [3]. The resulting problems include the need to triage [4] small resources over large areas and analyze great taxonomic diversity, as well as respond quickly to, vanishing opportunities for action.

An overwhelming proportion of tropical biodiversity is comprised of terrestrial arthropods, primarily insects. Spatial turnover in insect biodiversity occurs on a very small scale, comprising a data-rich and fine-grained source of biodiversity information. However, the paucity of trained arthropod taxonomists and the large number of species remaining to be described means that opportunities to utilize this information source are often missed. The presence of introduced and invasive species further complicates the study of arthropods. These newer arrivals make it difficult to differentiate newly introduced species from rare or unknown endemics. Further challenges include an abundance of cryptic species and inadequately-funded taxonomic research programs [5-7]. A database of standardized DNA sequences will empower researchers and land managers to predict and prevent the arrival of new species. Such planning could be used to avert potentially catastrophic effects [e.g. ecological meltdown - [8]].

Translocated species (i.e. invaders) are arriving at ever faster rates due to anthropogenic influence [9] and climate change [10], and biodiversity losses continue to accelerate [2]. Given these trends, can rates of species discovery and the enumeration of biodiversity keep pace? If limited to strictly traditional methods, science is almost certainly guaranteed to fall far behind. Taxonomy is an inherently difficult discipline requiring a lifetime of training. Established and formalized taxonomic frameworks exist only for relatively large, highly visible, and/or economically important vertebrates and arthropods. Too few taxonomists are available to survey the biodiversity of isolated or understudied areas, or to analyze the vast majority of terrestrial arthropods.

Taxonomists are acutely aware of these limitations. Many are now looking to use standardized DNA markers as DNA barcodes to address this problem [11,12]. Here, a gene (or genes) is collected into a publicly accessible genomics library using standardized methodologies. These involve comparing the barcode to sequence data from known species, as well as ancillary meta-data such as geography, observations and photographs. A regional query of such a database, based on the sequencing of a single specimen or environmental genomics using pyrosequencing technology [13], would allow researchers to compare diversity, uniqueness and complementarity at a far more rapid rate than morphological taxonomy alone.

Here we test the utility of a DNA barcoding approach to assess the diversity of understudied ant taxa on the tropical island of Mauritius. The flora and fauna of Mauritius have experienced 400 years of documented impacts from habitat modification and introduced species. We used 1111 specimens collected from 10 sites in 2005 to test whether DNA barcoding and traditional morphological taxonomic analyses would affirm the same units of diversity within and between these 10 sites. In addition, we examined whether rates of diversity and complementarity differed between standard morphological alpha-taxonomy or DNA barcoding. We tested whether the cost (here considered the loss of evolutionary history as measured by loss of barcode phylogenetic diversity - PD [14]) of a particular locality predicted the same relative importance of a locality. We further tested whether predictions for these estimates of evolutionary history were different between a mtDNA barcode region and a nuclear marker (28S, D2). We tested whether the relative importance of each site, or network of sites, (measured using the barcode alone), was affected by whether the specimen we had collected was known or presumed to be a native versus an introduced species. We do not attempt to provide a review of the criticisms of the efficacy of mitochondrial DNA barcoding [15-21], rather we highlight the importance of integrating ecological and historical information into biodiversity analyses that are based on DNA barcoding.

We conclude that merging DNA barcoding into diversity assessments allows researchers great opportunities to increase survey capacity. However, our study underscores the importance of tempering barcode analyses with natural history information, which help calibrate and improve the utility of this technique.

## Results

### Barcode Identification

**Figure 1:**
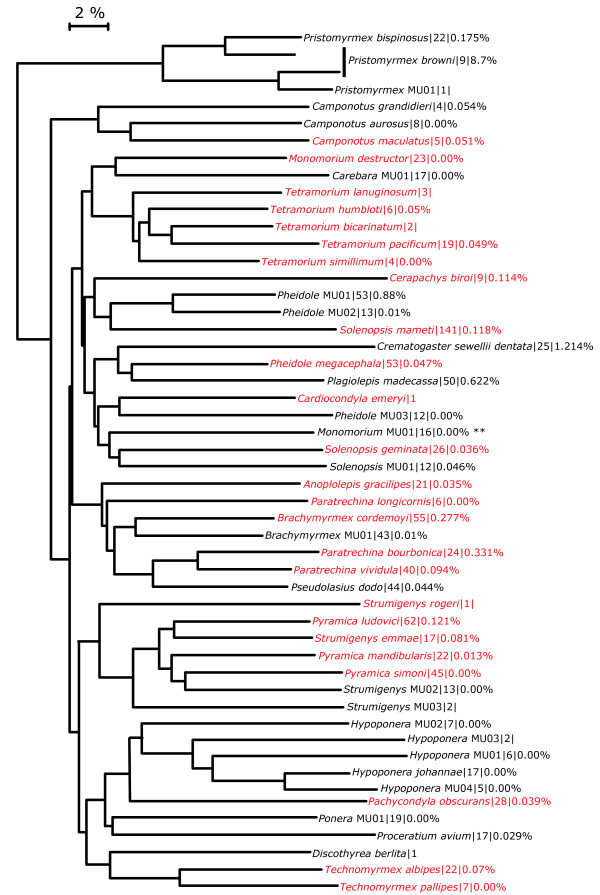
**Neighbor-joining tree of K2P distances for each of the 51 species of ants that were barcoded from Mauritius**. Only one representative of each species is shown. Branch tips are labeled as follows: species name (or provisional name when a formalized taxonomy has not been completed)|number of specimens sequenced for CO1|mean intraspecific divergence within the CO1 barcode region. Branch tips labeled in red are known introduced, or tramp species.

Fifty-one species were recorded from 165 collecting events across 10 localities (Figure 1). Specimens were identified to genus in Madagascar, and to morphospecies in San Francisco (USA) independent of barcoding. The species include a number of new records for the island [22].

**Figure 2:**
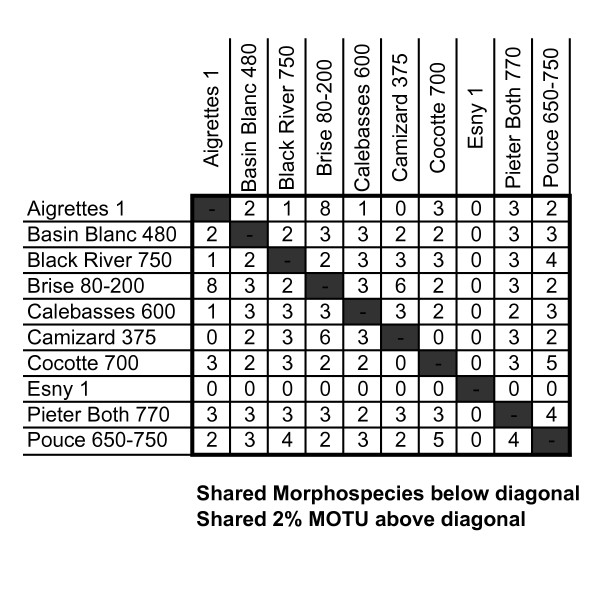
**Pairwise comparison of shared diversity between sites as measured by taxonomic richness (below diagonal) and molecular operational taxonomic units (or MOTU) using a 2% threshold (above the diagonal)**.

Barcode divergences (2%) used as a filter to compare to morphologically named units agreed in all cases except two (Figure 2). These two morphologically named taxa (*Hypoponera johannae *and *Pristomyrmex browni*) each contained much more than 2% sequence divergence (Table 1). Upon re-examination of the specimens we discovered sufficient morphological variation in the workers to justify classifying these specimens as candidate species. In addition, barcoding helped detect specimens that had been mislabeled or placed under the wrong species epithet. When sequences greater than 400 bp were compared, we found no significant departures from neutrality using Tajima's D (D = 1.10750, p > 0.10).

**Table 1:** Barcode divergence statistics (Minimum, Average and Maximum sequence divergence for CO1 sequences greater than 419 bp in length) for two apparent cases of morphologically cryptic variation.

Original Taxonomic Designation	Min	Average	Max	Provisional species following barcoding
*Hypoponera johannae*	0	18.571	23.825	*Hypoponera johannae* *Hypoponera sp. Mau-01* *Hypoponera sp. Mau-02* *Hypoponera sp. Mau-03* *Hypoponera sp. Mau-03*

*Pristomyrmex browni*	0	14.175	15.751	*Pristomyrmex browni* *Pristomyrmex sp. Mau-01* *Pristomyrmex sp. Mau-02*

In short, barcoding identified the same units of diversity (species) as were flagged using morphology (after deeply barcode divergent morphospecies were re-examined and re-allocated to new provisional species groups).

### Genetic Analyses and Divergent Intraspecific Lineages

Both rDNA markers used here (ITS1 and 28S) can help interpret morphologically cryptic and geographically sympatric deep mtDNA splits. As independent genetic markers, the correlated splitting of rDNA and mtDNA within a taxonomically ascribed single unit supports the hypothesis of morphologically cryptic species, while the lack of such a split can suggest mtDNA variation within a species, due to differences in rates of lineage sorting between mitochondrial and nuclear markers, hybridization between sister taxa or the presence of nuclear translocations of mitochondrial DNA [23-27].

#### Pristomyrmex

One *Pristomyrmex *morphospecies was collected from a critically threatened site (Le Pouce, [28]) through leaf-litter sampling and so represents an unknown number of colonies. We found deep divisions (15% CO1- Table 1) within this morphospecies, suggesting either that it contained multiple cryptic species, or that Le Pouce is a contemporary refuge for two apparently divergent mtDNA lineages. We tested whether these deep lineages were supported by nuclear markers. With 28S D2 (expected to be variable if two species), we found no variation. However, ITS1, expected to be hypervariable if two species, contained two clusters supporting CO1. All *Pristomyrmex *specimens tested positive for *Wolbachia*, and each provisional species harbored different species or numbers of infecting strains of *Wolbachia*. We hypothesize that these *Pristomyrmex *specimens are two recent or incipient species that have not yet accrued variation in the D2 region of 28S.

#### Paratrechina

rDNA variation within both *P. vividula *and *P. bourbonica *is not commensurate with barcode divergence or geography. An insertion (at ~100 bp) within the 28S D2 region of several sympatric *P. vividula *specimens may be representative of an rDNA pseudogene - or paralog. Paralogous sequences are a problem to specimen identification and comparison using either mtDNA [29], or rDNA [30,31]. Indeed, it can be difficult to identify rDNA pseudogenes. While protein coding mitochondrial genes can be checked for stop codons or translational errors, the non-coding regions of rDNA cannot. While a *Wolbachia *infection may explain the lack of mitochondrial variation within cases where there is apparent nuclear variation [32], we found no evidence of *Wolbachia *in any tested *Paratrechina *(Additional File 1). Since *P. vividula *is an introduced species, an alternative explanation for the apparently sympatric rDNA variation may lie in the fact that although the analysed *P. vividula *specimens are now sympatric, they may have originated from multiple founding populations. Further sampling is required to differentiate between the competing hypotheses of numt, paralogous and multiple founding populations as the source for this rDNA variation.

### Diversity Estimates

**Figure 3:**
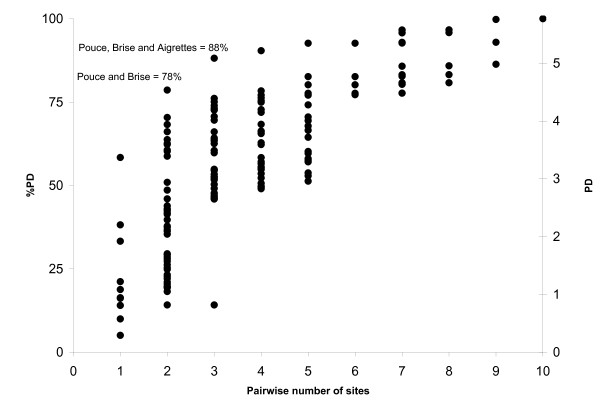
**Complement of diversity across sites**. Diversity (measured as percent Phylogenetic Diversity (PD)) as a function of pairwise comparisons across sites. Two sites (Brise and Le Pouce) contain nearly 80% of the genetic diversity sampled, while three sites (Brise, Le Pouce and Aigrettes) will include 88% of the total genetic diversity.

**Figure 4:**
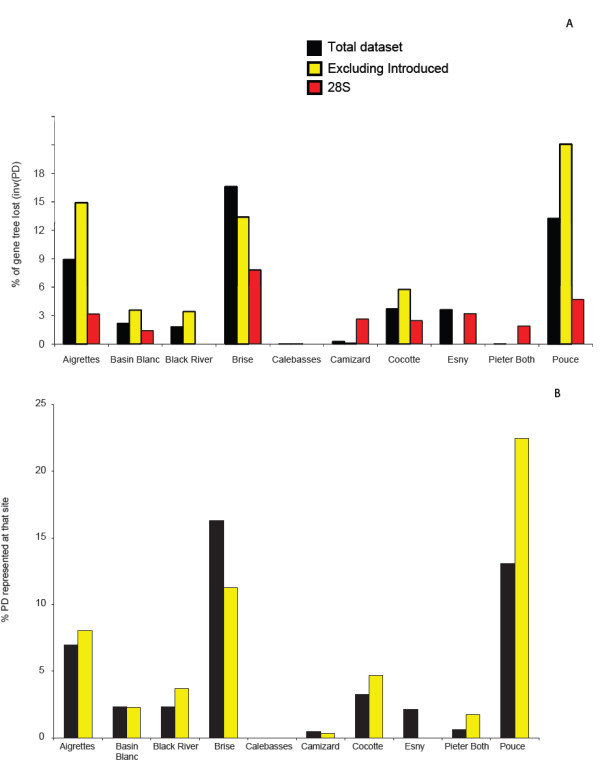
**A. Diversity (PD) represented within each site when the entire dataset is utilized (black bars) and when tramp species are not included (yellow bars)**. The rank importance of the three most diverse sites changes dramatically when known introduced species are excluded. B. The same analysis as in A except completed on a 135 bp tiny-barcode fragment from the extreme 5' end of the barcode region. Absolute values change, but the rank importance of each site and the effect of knowing whether species are native or tramp remain the same. This is the fragment size originally produced by pyrosequencing technology, suggesting that environmental barcode metagenomics of this hyperdiverse taxa would yield predictions equivalent to the sequencing technology used here (one sequence: one specimen).

Estimates of diversity (morphospecies richness, phylogenetic diversity [PD - sensu [14]] and barcode diversity ([essentially MOTU as in - [33]]]) were calculated for specimens from each of the 10 sites (Figure 3 &4). Using 1111 specimens (and all sequence lengths - i.e. not restricting analysis to the barcode convention of a minimum of 500 bp)) we found that the three most diverse sites, in descending order, were Brise, Le Pouce and Aigrettes. When barcode data were used in a PD approach to examine rarefaction (Figure 3) it became apparent that these three sites alone harbor nearly 90% of the total diversity collected on the island. The three least diverse sites were Pieter Both, Calabesses and Camizard, which contained primarily introduced species plus a low number of native species. Both MOTU and the morphospecies approach yielded almost identical estimates of complementarity for all ten sites (Figure 4).

Comparisons of diversity between localities were not altered by reducing the amplicon size or use of alternate genetic marker (Figure 4a). When barcode estimates of diversity (PD) were calculated using a truncated sequence length (130 bp), commonly produced by pyrosequencing technology in surveys of environmental metagenomics [13], there was no effect on between-site patterns (although because the total branch lengths in the tree are reduced, the total PD values are as well). Similarly, when a smaller number of specimens were compared using D2, the between-site patterns of diversity remained unchanged (Figure 4).

However, comparing between-site diversity using a restricted set of specimens that excluded known introduced species altered the site ranking. The three most diverse sites remained the same, but were ranked in a different order. By this measure, Le Pouce, an area with many endemic species relative to introduced species, was most diverse.

## Discussion

In the hyperdiverse ant fauna of Madagascar, and the relatively de-pauperate fauna of sub-arctic Canada, we have demonstrated [33,34] that DNA barcoding can accelerate current inventory methods and rapidly respond to pressing biodiversity needs. More specifically, this technique excels in the assessment of richness and turnover across landscapes. The initial Malagasy analysis involved a comparatively small number of sites (4) and specimens (268). The current study is larger in scale, including 10 sites and more than 1000 sequenced specimens (from collections of thousands of specimens from 165 collections). Even using an approach based on capillary sequencing (one specimen - one extraction - one sequence), DNA barcoding accelerates, and makes transparent and reproducible, our ability to estimate diversity and complementarity compared to morphology alone.

We found that barcode-based diversity estimates of PD were slightly but not significantly more dispersed than taxa-based (morphospecies) estimates. Furthermore, both the PD approach using barcodes and analyses based on morphology suggested that the same areas deserved protection. Unlike Forest et al., [35] who found that genetic and morphological measures of diversity were biased in different directions, our results suggest that identifying sites deserving protection based on a survey of barcode diversity yields the same results as would slower-to-generate morphological estimates. All methodologies agree that diversity is unevenly distributed throughout the ten study sites.

When introduced ants were excluded from the analyses, the diversity ranking changed. Localities judged to contain the most barcode diversity were affected by excluding species known to have been introduced species. For this reason, we place a high degree of importance on the biological, or natural history information associated with each specimen. Without this added information, genetic assessments of diversity can prioritize areas with artificially high diversity (due to introduced species) over areas with indigenous species that are fewer in number.

PD prediction-based trees built using a smaller amplicon (as might be used/produced in an environmental metagenomics survey) were not different from predictions using full length sequences (Figure 4b). This suggests that an environmental metagenomics approach, drawing on a reference library of full-length DNA barcodes, will provide rapid and information-rich estimates to triage conservation decisions.

Where tested, PD predictions of the barcode region were supported by the analysis of a non-mitochondrial marker. Not every barcoded specimen had an amplified nuclear marker, but, for those that did, patterns of within- and between- site diversity were the same whether obtained using morphospecies or barcode data (Figure 4a).

### Identification and Cryptic Diversity

*Pristomyrmex browni *specimens from Le Pouce were found to contain deep genetic divisions (15% CO1, 2 insertions in ITS1) while no differences were found within the D2 region of 28S (Table 1). We judged that these two genetic lineages were likely to be two provisional species living sympatrically, and that the nuclear marker 28S has had insufficient time to differentiate within the more conserved nuclear genome. In addition, genetically divergent strains of *Wolbachia *were found in specimens from each provisional species of ant. While related *Wolbachia *have been shown to infect related hosts, this association has not been demonstrated to extend to the species level [36]. As our analysis of *Wolbachia *infection is limited to the single *wsp *gene, and not the multi-locus MLST protocol [37], this result should be interpreted cautiously. However, further work may indicate that these provisional *Pristomyrmex *species represent a case where *Wolbachia *have spread with their host through co-divergence or introgression. The Le Pouce *Pristomyrmex *(originally identified as *P. browni *but discovered to be highly divergent using CO1 and other nuclear markers) provide an example of how integrating a standardized molecular marker into specimen surveillance can be more efficient than rapid provisional morphological identifications alone.

### Diversity Estimates

The island of Mauritius was originally entirely covered by dense forest. However, most forests have been logged since human colonization approximately 400 years ago. The forest patches that remain are surrounded and infiltrated by numerous introduced animal and plant species.

The known native ant fauna of Mauritius currently includes 18 native species, 9 of which are endemic to the island [22]. All surveys to date indicate endemic ants are confined to upland forest on mountaintops. These endemics could be the only remaining examples of a much richer endemic fauna that disappeared with the destruction of the lowland forest. The recent discovery of a new genus record on Le Pouce [22] strongly suggests that even more species await discovery on the island.

Le Pouce is an apparent sanctuary of taxonomically peculiar endemic ant species [22]. Of the ten sites surveyed here, it is one of the two most genetically diverse [PD - sensu [14]] on the island (Figure 4). When tramp species were included in the analysis, we found that the most PD diverse site is Brise, which contained both a large complement of introduced species and also a relatively large number of native and endemic species. Ranking sites by genetic diversity, including and excluding introduced species, suggests that Le Pouce and Brise warrant the greatest degree of conservation. Only 2 MOTU or morphospecies are common to both sites. Brise may be farther along the invasion progression that threatens native populations. Because Le Pouce populations are located at higher elevations (700-800 m versus 200 m for Brice) they may be less susceptible to invasion from introduced species. Conservation efforts should be directed at protecting both remaining populations while they are still healthy.

The ability to estimate the genetic diversity of a site or series of locations will likely become standard practice when eukaryotic environmental genomics becomes more commonplace and affordable. Accordingly, we tested whether our conclusions were altered by reducing the sequence comparison from full length barcode region to the truncated sequence length originally produced by pyrosequencing technology. This '*in silico' *test region corresponds to the same small region used previously to test the effect of a minimalist barcode on species identification [38,39].

This work adds to the growing body literature demonstrating that PD in general provides a unique and important measure of biological diversity [40], and further that PD estimates based on standardized DNA barcodes will provide a critical scaffold for comparing those estimates between taxa and sites [34].

### Invasions

*"Obviously unless something is done soon to stem the invasion of exotic species, the indigenous forests of Mauritius will face extinction." *[[41] - p. 161.]

Used as a standard first-pass approach, DNA barcodes will permit genetic estimates of diversity to be applied in a range of biodiversity and conservation projects. Barcoding permits much faster estimates of diversity and complementarity, and is generated in a fashion that permits easier comparisons between research programs and taxa. Our work demonstrates that using a PD approach for these standardized sequences generates measures of diversity equivalent to morphological estimates. At the same time, it permits researchers to make hypotheses regarding whether divergent/discontinuous barcode diversity is equivalent to a unique species. Furthermore, our work demonstrates the importance of knowing the organism. We find that unless the introduced or native status of specimens is known, an exclusively genetic approach to diversity and site protection may be biased towards sites that have higher rates of established introduced species rather than higher rates of native diversity.

## Conclusion

Ant diversity is known to be very sensitive to environmental variables such as the presence of leaf-litter and soil type [42], and to change over small spatial scales [43,44]. This combination could provide information-rich estimates of biodiversity [45], endemism, and population isolation and viability. However, species-level insect identification can be notoriously difficult [46,47], dependent on specific life-history stages for positive identification [48], complicated by numerous synonymies [49], and likely overlooks many cryptic species [25-27]. We have shown here that integrating a first-pass [50] CO1 DNA barcode approach will permit far more rapid estimates of diversity and complementarity than morphological analysis alone. These predictions were resilient to length of amplicon size and not significantly different from PD estimates using a nuclear marker. Critically, the information was best interpreted when knowledge of the natural history of the animal was overlaid onto the patterns of genetic diversity (e.g. the inclusion or exclusion of known tramp species can affect the ranking of sites for conservation). Integrating DNA barcoding in a collaborative effort to rank sites rapidly based on diversity will yield results with high discriminatory power, transparency and reproducibility to the benefit of science and conservation.

## Methods

### Collection

This work is based on ant inventories in Mauritius conducted from 25 May-31 May, 2005. During that period, one of us (BLF) and a team of four experienced Malagasy ant collectors visited ten sites: Le Pouce Mt., Pieter Both Mt., and Calebasses Mt. in the Moka Range; Camizard Mt., and Brise Mt. in the Bambous Range; and Basin Blanc, Ile aux Aigrettes, Point D'Esny, Cocotte Mt., and Petite Rivière Noire Mt. Ants were collected using general hand-search techniques and leaf litter extraction.

### Molecular

Total genomic DNA extracts were prepared from small pieces (≤ 1 mm) of tissue using the NucleoSpin^® ^96 Tissue kit (Macherey-Nagel Duren, Germany) following the manufacturer's protocols. Extracts were resuspended in 30 μl of dH2O, and a 650 base-pair (bp) region near the 5' terminus of the CO1 gene was amplified following standard protocols [51-53].

Extracts were resuspended in 20-30 μl of dH2O. A 658 region near the 5' terminus of the CO1 gene was amplified using primers LepF1/LepR1. In cases where a full length product was not successfully generated, internal primer pairs (LepF1/C_ANTMR1D) and (MLepF1/LepR1) were employed to generate shorter sequences. These could be overlapped to create composite sequence (contig) or could be analyzed as shorter, non-barcode-standard length standard sequences. (See Table 2 for a complete list of primers and sources).

**Table 2:** Primers used to generate sequences and molecular tests.

Primer Name	Primer sequence (5'-3')	Amplicon region	Primer source	Used for sequencing (Y/N)
LepF1	ATTCAACCAATCATAAAGATATTGG	CO1	[66]	Y

LepR1	TAAACTTCTGGATGTCCAAAAAATCA	CO1	[53]	Y

MLepF1	GCTTTCCCACGAATAAATAATA	CO1	[67]	Y

C_ANTMR1D-RonIIdeg_R	GGRGGRTARAYAGTTCATCCWGTWCC	CO1	[Modified from [68]]	N

C_ANTMR1D-AMR1deg_R	CAWCCWGTWCCKRMNCCWKCAT	CO1	[Modified from [33]]	N

D2B	GTCGGGTTGCTTGAGAGTGC	28S	[69]	Y

D3Ar	TCCGTGTTTCAAGACGGGTC	28S	[69]	Y

CAS18Fs1	TACACACCGCCCGTCGCTACTA	ITS1	[70]	Y

CAS5p8s1Bd	ATGTGCGTTCRAAATGTCGATGTTCA	ITS1	[Modified from [70]]	Y

*wsp *81F	TGGTCCAATAAGTGATGAAGAAAC	*Wolbachia *surface protein	[60]	Y

*wsp *691R	AAAAATTAAACGCTACTCCA	*Wolbachia *surface protein	[60]	Y

PCR reactions were carried out in 96 well plates in 12.5 μl reaction volumes containing: 2.5 mM MgCl_2_, 1.25 pmol of each primer, 50 μM dNTPs, 10 mM Tris HCl (pH 8.3), 50 mM KCl, 10-20 ng (1-2 μl) of genomic DNA, and 0.3 unit of TaqDNA polymerase (Platinum^® ^Taq DNA Polymerase - Invitrogen) using a thermocycling profile of one cycle of 2 min at 94°C, five cycles of 40 sec at 94°C, 40 sec at 45°C, and 1 min at 72°C, followed by 36 cycles of 40 sec at 94°C, 40 sec at 51°C, and 1 min at 72°C, with a final step of 5 min at 72°C. Products were visualized on a 2% agarose E-Gel^® ^96-well system (Invitrogen) and samples containing clean single bands were bidirectionally sequenced using BigDye v3.1 on an ABI 3730xl DNA Analyzer (Applied Biosystems).

Contigs were made using Sequencher v4.0.5 (Gene Codes) and the Contig Express module of Vector NTI v10 (Invitrogen Corp.) and subsequently aligned by eye in Bioedit [54]. Sequence divergences were calculated using the K2P distance model [55] and a NJ tree of distances [56] was created to provide a graphic representation of the patterning of among-species divergences using MEGA4[24], and BOLD [57]. Tests for sequence neutrality [Tajima's D - [58]] and rates of substitution were calculated with DNAsp [59].

Sequences, trace files and other specimen information are available in the project file "Ant Diversity of Mauritius [ASMA]" in the Published Projects section of the Barcode of Life website http://www.barcodinglife.org with complete collection information for each specimen deposited at http://www.antweb.org. All sequences from the barcode region have been deposited in GenBank [EF609645-EF610627, EU150286-EU150369 &EU525187-EU525240].

### Complementary genetic analyses

In addition to the CO1 barcode region, we amplified portions of the rDNA gene regions for a portion of the large subunit (LSU or 28S - variable D2 region) for 206 specimens and the variable spacer region (ITS1) for 51 specimens. Specimens selected for this complementary treatment had displayed one of two features on initial analysis. In the first case, their initial barcode analyses had demonstrated large mitochondrial divergences within single morphological ascribed units. Alternatively, the initial barcode analysis had, 'failed' in that no barcode was produced and in this case we tested the validity of the re-extraction using ITS1 or 28S rDNA. The 28S amplicon forward primer corresponds to positions 3549-3568 in the *Drosophila melanogaster *reference sequence (GenBank M21017). The ITS1 forward primer used corresponds to positions 1822-1843 in the same *D. melanogaster *reference sequence. Primers used to generate these fragments are listed in Table 2. Representative sequences have been deposited in GenBank: [28S: EU401992-EU402079, EU417909-EU417943, EU439628-EU439648 &EU490435-EU490496; and ITS1: EU439616-EU439627, EU518129-EU518168].

For nearly a third of the specimens barcoded, we utilized a standard PCR diagnostic to test for the presence of *Wolbachia *[60]. *Wolbachia *are obligate intracellular endosymbiotic bacteria that cause reproductive incompatibility between infected and uninfected lineages, resulting in an increased proportion of infected maternal lineages that cannot reproduce [61]. The assay we utilized is a PCR-based test for a *Wolbachia *specific surface coding protein (wsp). As the extracts tested are generally from ant legs, the *Wolbachia *presence/absence test should be considered conservative (i.e. since reproductive organs were not extracted, less severe infections would not likely yield a positive reaction in this test, and would thus constitute false negatives). In addition to this selective assay, we observed 6 cases where initial barcode amplification from *Plagiolepis madecassa *DNA extracts resulted in CO1 amplicons of *Wolbachia*. In each case, these amplicons were identified as bacterial contaminants and excluded from analyses of Formicidae. Subsequent re-amplification produced the *P. madecassa *CO1. *Wolbachia *WSP sequences from *Pristomyrmex browni, Plagiolepis madecasa, Pheidole megacephala, Technomyrmex albipes, Strumigenyis *MU02, and *Pyramica ludovici *have been deposited in GenBank [EU5181169-EU518183].

Rarefaction curves were generated for pairwise combinations of study localities using the program CONSERVE IV (version v1.3).

See Additional File 1, for all collection information, sequence information, GenBank accessions, *Wolbachia *test results and specimens accessions for specimens used here.

### Diversity

We tested whether the pairwise comparison of locality diversity was affected by measuring biodiversity using morphology or DNA based units of diversity.

Indices based on sampling the genetic diversity of taxa and areas have been proposed to standardize and increase the rate of sampling localities and to provide a more accurate reflection of evolutionary history than morphological analyses alone [62-64]. To test this hypothesis, we created neighbor-joining trees (K2P distances) for all specimens included here with CO1 sequences longer than 500 bp. We then utilized the program CONSERVE [65] to determine the proportion of phylogenetic diversity (as an estimate of evolutionary history [14]) reflected in the barcode region maintained in that geographic location. We completed pair-wise comparisons of all combinations for the ten sites to see what minimum number of localities preserved the most genetic diversity on the island. This type of genetic analysis of biodiversity was completed on specimens 1) with truncated sequence length (130 bp); 2) for which we had also sequenced 28S D2 rDNA; and 3) coded by site and whether they were known to be native or introduced species on Mauritius. The short fragment used here is comparable to the short universally-primed amplicon proposed to be ideal for sequence characterization environmental mixtures through massively parallelized sequencing technologies [39].

## Competing interests

The authors declare that they have no competing interests.

## Authors' contributions

BLF carried out the field work and morphological studies, MAS carried out the molecular genetic studies, diversity analyses and drafted the manuscript. Both authors conceived of the study, and participated in its design and coordination, helped to draft the manuscript and have read and approved the final manuscript.

## Authors' information

MAS is a molecular ecologist at the Biodiversity Institute of Ontario and the Department of Integrative Biology at the University of Guelph, Ontario, Canada whose research involves species delineation, diversity assessment, conservation genetics and population isolation. BLF is the Chairman of Entomology at the California Academy of Sciences, San Francisco, USA and researches ant taxonomy, conservation biology and species delineation.

## Supplementary Material

Additional file 1 **Relevant information for all specimens included in this manuscript**. CO1 Process ID, Sample ID, Field Num, CO1 Seq Length, CO1 Genbank, 28S Genbank, ITS1 Genbank, WSP+/-, SP Genbank, Collection Date, Identification, Extra Info, Genus, Species, Identifier, Identifier email, Collectors, Date Collected, Country, Region, Sector, Exact Site, Lat, Lon, Elev, Notes. Click here for file
